# Ceftriaxone use in a tertiary care hospital in Kilimanjaro, Tanzania: A need for a hospital antibiotic stewardship programme

**DOI:** 10.1371/journal.pone.0220261

**Published:** 2019-08-05

**Authors:** Tolbert B. Sonda, Pius G. Horumpende, Happiness H. Kumburu, Marco van Zwetselaar, Stephen E. Mshana, Michael Alifrangis, Ole Lund, Frank M. Aarestrup, Jaffu O. Chilongola, Blandina T. Mmbaga, Gibson S. Kibiki

**Affiliations:** 1 Kilimanjaro Clinical Research Institute, Kilimanjaro Christian Medical Centre, Moshi, Tanzania; 2 Department of Biochemistry and Molecular Biology, Kilimanjaro Christian Medical University College, Moshi, Tanzania; 3 Lugalo General Military Hospital, Military College of Medical Sciences, Dar es Salaam, Tanzania; 4 Catholic University of Health and Allied Sciences, Mwanza, Tanzania; 5 Centre for Medical Parasitology, Copenhagen University Hospital, Copenhagen, Denmark; 6 Centre for Biological Sequence Analysis, Technical University of Denmark; Copenhagen, Denmark; 7 Centre for Genomic Epidemiology, Technical University of Denmark; Copenhagen, Denmark; 8 The East Africa Health Research Commission, Bujumbura, Burundi; University of the Witwatersrand, SOUTH AFRICA

## Abstract

Excessive use of antibiotics, especially watch group antibiotics such as ceftriaxone leads to emergence and spread of antimicrobial resistance (AMR). In low and middle-income countries (LMICs), antibiotics are overused but data on consumption is scarcely available. We aimed at determining the extent and predictors of ceftriaxone use in a tertiary care university teaching hospital in Kilimanjaro, Tanzania. A hospital-based cross-sectional study was conducted from August 2013 through August 2015. Patients admitted in the medical, surgical wards and their respective intensive care units, receiving antimicrobials and other medications for various ailments were enrolled. Socio-demographic and clinical data were recorded in a structured questionnaire from patients’ files and logistic regression was performed to determine the predictors for ceftriaxone use. Out of the 630 patients included in this study, 322 (51.1%) patients were on ceftriaxone during their time of hospitalization. Twenty-two patients out of 320 (6.9%) had been on ceftriaxone treatment without evidence of infection. Ceftriaxone use for surgical prophylaxis was 44 (40.7%), of which 32 (72.7%) and 9 (20.5%) received ceftriaxone prophylaxis before and after surgery, respectively. Three (6.8%) received ceftriaxone prophylaxis during surgery. Predicting factors for that the health facility administered ceftriaxone were identified as history of any medication use before referral to hospital [OR = 3.4, 95% CI (1.0–11.4), p = 0.047], bacterial infection [OR = 18.0, 95% CI (1.4–225.7, p = 0.025)], surgical ward [OR = 2.9, 95% CI (0.9–9.4), p = 0.078] and medical wards [OR = 5.0, 95% CI (0.9–28.3), p = 0.070]. Overall, a high ceftriaxone use at KCMC hospital was observed. Antimicrobial stewardship programs are highly needed to monitor and regulate hospital antimicrobial consumption, which in turn could help in halting the rising crisis of antimicrobial resistance.

## Introduction

Ceftriaxone is a third generation cephalosporin antibiotic. It is among a group of broad-spectrum antibiotics covering a wide range of infections. It is used as a first choice for acute bacterial meningitis, community acquired pneumonia (severe), complicated intra-abdominal infections (mild to moderate), complicated intra-abdominal infections (severe), hospital acquired pneumonia, *Neisseria gonorrhoeae*, pyelonephritis or prostatitis (severe). It is used as second choice for acute invasive bacterial diarrhoea / dysentery, bone and joint infections, pyelonephritis or prostatitis (mild to moderate), sepsis in neonates and children. [1,2].

In Tanzania, as in many other countries, ceftriaxone is as well among the “watch group” antibiotics[3–5]. Cephalosporins should only be prescribed when there is evidence of infection such as increase in serum procalcitonin levels or bacterial culture and sensitivity results from the clinical laboratory. However, ceftriaxone has often inappropriately and excessively been prescribed in clinical settings especially where there is lack of clear diagnosis[6,7]. Although ceftriaxone can be used as prophylaxis in certain situations, in a National Hospital in Tanzania, ceftriaxone was the most given prophylactic antibiotic regardless of the urological surgery done and its level of contamination[8]. The impact of irrational use of ceftriaxone on development of resistance to third-generation cephalosporins among clinical strains of *Enterobacteriacea* and other non- enteric bacteria is well known[9–11] and is one of the emerging global public health issues, particularly in LMICs [7,12]. This has furthermore lead the World Health Organization (WHO) to call for optimization of antimicrobial use to curb AMR [13]. If resistance to ceftriaxone is becoming widespread, not only are very few alternative antibiotic options available in LMICs like Tanzania, but these few options are as well unaffordable by the majority patients.

Unfortunately, resistance to third generation cephalosporins is already very high. For instance, in one review on AMR covering Eastern African countries the proportion of Gram negative and positive bacteria that were resistant to ceftriaxone was ranging from 46–96% and 50–100%, respectively[14]. In northwestern Tanzania, resistance to ceftriaxone was 29.4% in 2010[15] and 35% (in 2014) regarding resistance to carbapenems[16]. Furthermore, a study from Mwanza, Tanzania observed that 25 (80.6%) of *Klebsiella pneumoniae* were cephalosporin resistant and overall increase in resistant isolates to third-generation cephalosporins rose from 26.5% in 2014 to 57.9% in 2015 [17]. Another study from a tertiary care, university teaching hospital in Mwanza, Tanzania had previously revealed an increasing trend of bacterial resistance against ceftriaxone from 14% in 2009 to 29.4% in 2011[15]. In a 2013–2015 study at a tertiary care hospital in Kilimanjaro the reported resistance to ceftriaxone by Gram-negative bacteria among in-patients was 51.8% [18]. In East Africa, including Tanzania, there is a paucity of timely data on antibiotic consumption especially on ceftriaxone use in hospitals[14,19]. Therefore, the present study aimed at identifying the extent of ceftriaxone use and determining predictors of ceftriaxone use at a tertiary care and a university teaching hospital in Kilimanjaro Tanzania.

## Materials and methods

### Ethical approval and participant’s consent

This study was granted ethical approval by the KCMC Research Ethics Committee and the National Institute for Medical Research with approval numbers 893 and NIMR/HQ/R.8a/Vol.IX/2080 respectively. A written informed consent was obtained from each participant or from parents or guardians of children before enrolment into the study.

### Study settings and design

A cross-sectional study was carried out from August 2013 through August 2015 at Kilimanjaro Christian Medical Centre (KCMC). KCMC is a consultant, tertiary care and a referral hospital located in Moshi municipality. It has a 650-bed capacity and the second largest consultant referral university teaching hospital serving over 12 million people from northern and central regions of Tanzania (http://www.kcmc.ac.tz/). The study involved inpatients. All admitted patients in medical and surgical wards and their respective Intensive Care Units who had a documented presumptive diagnosis of septicaemia and upper respiratory tract infection were enrolled. Also enrolled were those patients with diarrhoea, diabetic ulcer, patients with fever of unknown cause, wounds due to burns, surgical procedures, diabetes mellitus, animal bites, motor traffic accidents and other injuries. The study was granted ethical approval by the KCMC Research Ethics Committee and the National Institute for Medical Research. A written informed consent was obtained from each participant or from parents or guardians of children before enrollment into the study.

### Data abstraction and analysis

Data were extracted from patient files among all inpatients in medical and surgical wards with a presumptive diagnosis of a bacterial infection. Data collected include type of ward, use of ceftriaxone or any other antibiotics, clinical diagnoses and socio-demographics. All data were recorded in a structured questionnaire and double entered in OpenClinica (OpenClinica LLC, MA, USA). Data were cleaned and analyzed using Stata 13 (StataCorp LP, Texas 77845, USA). The proportion of ceftriaxone use was determined by dividing the number of patients taking ceftriaxone by the total number of patients who had a presumptive diagnosis of a bacterial infection. The prevalence of ceftriaxone use across categorical variables (such as gender, timing of prophylaxis, presence of infection, diagnoses, number of days of hospital stay et cetera) was compared using Chi-square or Fisher’s exact tests. Bivariate and adjusted logistic regression analyses were used to determine possible factors that were associated with ceftriaxone use. Both forward selection and backward elimination model building approaches were performed and were both found to predict the same final model. Statistical significance was set at cut off points of 0.20 and 0.10 for bivariate and adjusted analyses respectively.

## Results

### Study population characteristics

The study population included patients admitted in the wards of KCMC as previously described by Kumburu et al[18]. A total of 630 patients were included in analysis, of which males were 360 (59.1%), 343 (58.8%) were married and 256 (42.6%) were aged 19–45 years. Those with primary education were 359 (62.1%), farmers were 290 (49.2%) and who had stayed in hospital for a week or less were 393 (64.0%) (Table 1).

**Table 1:** Study population characteristics.

Characteristics	n (%)
Age group (years)	
< = 18	76 (12.1)
19–45	256 (40.6)
46–65	182 (28.9)
66+	87 (13.8)
Missing	29 (4.6)
Gender	
Female	249 (39.5)
Male	360 (57.2)
Missing	21 (3.3)
Education	
No formal education	108 (17.2)
Primary	359 (57.0)
Secondary	74 (11.6)
Tertiary	37 (5.9)
Missing	52 (8.3)
Marital status	
Single	168 (26.7)
Married	343 (54.4)
Widowed	47 (7.5)
Divorced	25 (3.4)
Missing	47 (7.5)
Occupation	
Peasantry	290 (46.0)
Employed	59 (09.4)
Business	125 (19.8)
Others	116 (18.4.)
Missing	40 (6.4)
Hospital stay (days)	
≤7	393 (62.4)
8–14	105 (16.7)
≥14	116 (18.4)
Missing	16 (2.5)

### Prevalence of ceftriaxone use

Out of the 630 patients, 322 patients (51.1%) were on ceftriaxone during their time of hospitalization. Out of 108 patients who underwent surgery, 44 (40.7%) received ceftriaxone for surgical prophylaxis. Ceftriaxone prophylaxis before and after surgery was given in 32 (72.7%) and 9 (20.5%) patients, respectively. Three (6.8%) received ceftriaxone prophylaxis during surgery. Twenty-two (6.9%) of the patients received ceftriaxone treatment without having any infection as determined clinically. A total of 166 patients (51.5%) with wounds received ceftriaxone as treatment of choice. 126 (39.8%) and 81 (25.6%) of patients admitted in surgical and medical wards, respectively received ceftriaxone during their treatment. The possible association between period admitted at hospital and ceftriaxone use was evaluated: Among patients with ≤ 7 days admitted, 195 (61.9%) were on ceftriaxone, while patients admitted for 8–14 days it was 66 (21.0%) and patients admitted for more than 14 days it was 54 (17.1%) (Table 2).

**Table 2:** Ceftriaxone use among inpatients at KCMC hospital.

Characteristic	n (%)	χ^2^	p
Over all	322 (51.1)		
Gender			
Female	137 (43.2)	1.48	0.223
Male	180 (56.8)		
Surgical Prophylaxis			
Before surgery	32 (72.7)	6.02	0.049
During surgery	3 (6.8)		
After surgery	9 (20.5)		
Infection present			
No	22 (6.9)	5.89	0.015
Yes	298 (93.1)		
Diagnoses			
cellulitis			
No	308 (95.7)	5.27	0.022
Yes	14 (4.3)		
cough			
No	318 (98.8)	0.004	0.95
Yes	4 (1.2)		
diabetes			
No	286 (88.8)	5.72	0.017
Yes	36 (11.2)		
wound			
No	156 (48.4)	11.15	0.001
Yes	166 (51.5)		
meningitis			
No	317 (98.4)	2.59	0.107
Yes	5 (1.6)		
diarrhoea			
No	318 (98.7)	7.08	
Yes	4 (1.3)		
septicaemia			
No	294 (91.3)	2.97	0.085
Yes	28 (8.7)		
pneumonia			
No	286 (88.8)	0.2	0.652
Yes	36 (11.2)		
Ward type			
Surgical1			
No	191(60.2)	10	0.002
Yes	126 (39.8)		
Surgical2			
No	306 (96.5)	0.28	0.597
Yes	11 (3.5)		
Surgical ICU			
No	300 (94.6)	2.21	0.137
Yes	17 (5.4)		
Medical 1			
No	236 (74.4)	2.03	0.154
Yes	81 (25.6)		
Medical 2			
No	260 (82.0)	1.18	0.277
Yes	57 (18.0)		
Medical ICU			
No	298 (94.0)	4.18	0.041
Yes	19 (6.0)		
Other wards			
No	311 (98.1)	19.2	0
Yes	6 (1.9)		
Hospital stay (days)			
≤ 7	195 (61.9)	7.1	0.029
01/08/14	66 (21.0)		
Above 14	54 (17.1)		

### Factors predicting ceftriaxone use among inpatients at KCMC hospital

**Fig 1:**
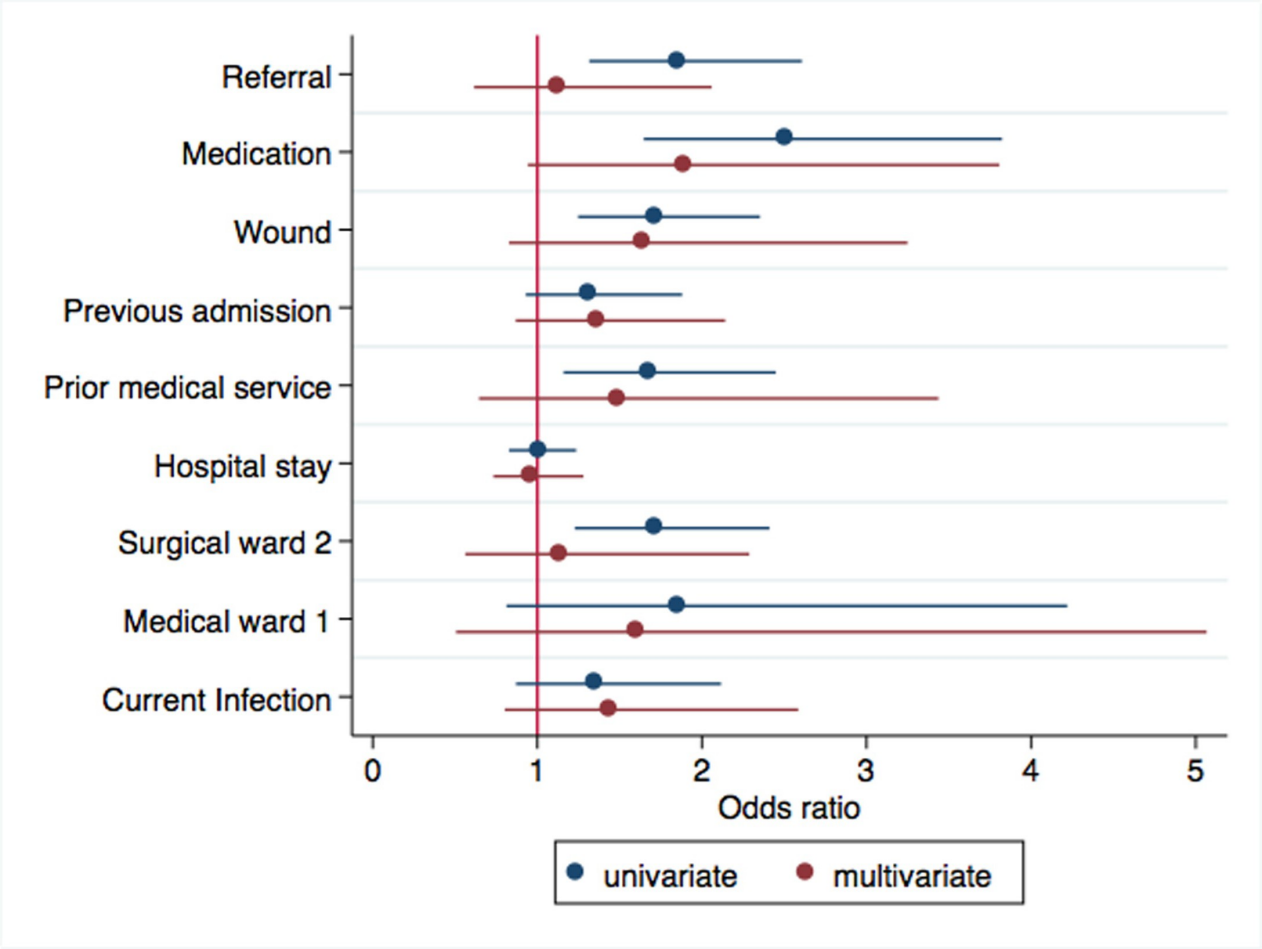
A forest plot to show univariable and multivariable regression analysis on factors predicting ceftriaxone use. Referral (Transferred from another hospital), Medication (Patient on any medication), Wound (Any wound, including septic infected burn wound), Previous admission (Previous admission to hospital), Prior medical service (Any medical service before coming to hospital), Hospital stay (Length of hospital stay in days), Surgical ward 2 (Department of surgery ward 2), Medical ward 1 (Department of Medicine ward 2), Current Infection (A Presumptive diagnosis of infection present at admission).

Bivariate and multivariable regression analyses were performed to identify predictors of ceftriaxone use in KCMC hospital. Significant predictors of ceftriaxone use on a bivariate level were: transfer from another hospital [OR = 1.9, 95% CI (1.3–2.6), p = 0.000], all wound types [OR = 1.7, 95% CI (1.3–2.4), p = 0.001], seeking or looking for any medical service before coming to hospital [OR = 1.7, 95% CI (1.2–2.5), p = 0.006], history of medication use before coming to hospital [OR = 2.5, 95% CI (1.7–3.8), p = 0.000], the patient having any bacterial infection [OR = 2.0, 95% CI (1.1–3.4), p = 0.017], the patient being under any medication [OR = 14.4, 95% CI (1.9–110.7), p = 0.011], septic infected burn wound [OR = 1.8, 95% CI (1.0–3.2), p = 0.041], diarrhoea [OR = 0.3, 95% CI (0.1–0.8), p = 0.014] and surgical 1 ward [OR = 1.7, 95% CI (1.2–2.4), p = 0.002]. Factors that were significantly associated with ceftriaxone use after adjusting for other factors include history of medication use before coming to hospital [OR = 3.4, 95% CI (1.0–11.1), p = 0.047], the patient having any bacterial infection [OR = 18, 95% CI (1.4–225.7), p = 0.025], Surgical ward 2 [OR = 2.9, 95% CI (0.9–9.4), p = 0.078] and Medical ward 1 [OR = 5.0, 95% CI (0.9–28.3), p = 0.070] (Fig 1)

## Discussion

This study was carried out to investigate ceftriaxone use among inpatients in a tertiary care university teaching hospital in Kilimanjaro, Tanzania. The overall use of ceftriaxone among inpatients in this study was high (51.1%). Ceftriaxone is among the most commonly utilized antibiotics owing to its high potency, a wide spectrum of activity, and a low risk of toxicity[5]. It is used to treat different types of bacterial infections including pneumonia, bone, abdominal, skin and soft tissue, and urinary tract [5][20]. It has an advantage of a wide coverage of pathogens, easy administration as it is once daily dosing–limiting nursing time needed and a low cost compared to many antibiotics.[21] [22][23] Other studies have as well shown a relatively similar extent of use to the current study; for instance, in a study from a tertiary care hospital in Dar es Salaam where ceftriaxone use as urological surgical prophylaxis was 46.1% [8] and in Nigeria ceftriaxone use for a suspected systemic infection was 42.3% [24] and even higher use in Ethiopia empiric ceftriaxone use was 58% [25] presumptive diagnoses of pneumonia, meningitis and sepsis led to ceftriaxone use in 59.3% [12] and the utilization rate of ceftriaxone was found to be high with a point prevalence of 59% where ceftriaxone was empirically used in 79.5% of cases [5]. Thus, generally, the observed ceftriaxone use in the present study as is in other studies is irrationally high as this drug (and as well, other cephalosporins) should only be used as a life saving drug against confirmed severe infections and not prescribed routinely. Another indication for cephalosporins should be in confirmed resistance to first and second line antibiotics. Nonchalant ceftriaxone prescription habits by Tanzanian clinicians has been observed to be a common practice in many clinical settings[8] which warrants immediate intervention through antimicrobial stewardship that can monitor adherence to prescription guidelines.

In suspected infection clinicians are not entirely sure if the offending organism is a bacterium. Other causes of fever may be viruses and fungi or even dehydration. In some circumstances it is advised to not use any antibiotic as the causative organism could be a virus[26]. Alternatives to ceftriaxone that should be suggested instead include fortified penicillin such as amoxicillin clavulanate; macrolides like azithromycin; and aminoglycosides like gentamicin depending on the offending bacteria infection [27].

In the present study, ceftriaxone use for surgical prophylaxis was 40.7% and importantly, out of these, 20.5% received ceftriaxone prophylaxis after surgery. This is a prolonged antibiotic prophylaxis constituting an unnecessarily excessive antibiotic exposure, which is an important driver of AMR emergence and spread. Similar observations were seen from the hospital in Dar es Salaam regarding use of ceftriaxone for prophylaxis where 86.4% of patients were put on post operative ceftriaxone prophylaxis for up to five days in the majority of elective, clean surgeries[8] and for surgeries involving thyroidectomy (58.2%) and cystectomy (47.8%) [28]. Prolonged surgical ceftriaxone prophylaxes are also observed in a tertiary care hospital in Ethiopia where (94%) had post operative prophylaxis and (70%) used ceftriaxone [29]. Again in Ethiopia ceftriaxone surgical prophylaxis was (84.5%)[30]. The absence of local surgical prophylactic guidelines was identified to be the cause of excessive ceftriaxone administration. Other documented causes are absence of first and second generation cephalosporins, low cost of ceftriaxone, absence of microbiologic data to inform both prophylactic and empiric antibiotic practice and lack of evidence based protocol for the settings where ceftriaxone are over administered[29]. Intuitively, some clean surgeries do not even need antibiotic prophylaxes. Alternative agents to ceftriaxone prophylaxis, according to The *Scottish* Intercollegiate Guidelines Network (SIGN) guidelines, depending on surgery types are penicillins e.g. benzylpenicillin for suspected streptococci, N. meningitidis, and spirochaetes; Aminoglycosides e.g. gentamicin mostly for suspected Gram-negative bacteria including Pseudomonas; Macrolides e.g. azithromycin for suspected Gram-positive bacteria and some Gram-negative bacteria (Haemophilus, Neisseria, Moraxella), has an important activity against intracellular bacteria including Chlamydia, Legionella, Mycoplasma species and to patients allergic to beta lactams; Fluoroquinolones e.g. ciprofloxacin for suspected Gram positive and Gram-negative bacteria including Pseudomonas; Nitroimidazoles e.g. metronidazole for suspected anaerobes[27].

The present study as well documents a low level (6.9%) of ceftriaxone use without evidence of infection (empiric treatment) which is much lower compared to a study in Uganda with a 77.7% empiric ceftriaxone use among children suspected of central nervous system infection[31]. One might argue that the central nervous system infection may be quite severe and other antibiotics might be resistant in Ugandan children, hence the choice of ceftriaxone. Other studies in Ethiopia indicated ceftriaxone empiric treatment to range between 79.0–87.3% [5,25]. While such ceftriaxone prescribing practices may be life saving, a need arises to be guided by locally generated data on sensitivity patterns[32–35]. To rationalize antimicrobial consumption and mitigate rapid development of AMR, hospital antimicrobial stewardship programs should impose and enforce prescription restrictions, set up antibiotic consumption surveillance systems and deliver appropriate educational campaigns to prescribers. Stewardship will as well increase knowledge on resistance patterns to secure other low spectrum antibiotics that can still be effectively used. There is evidence showing a significant reduction in ceftriaxone use from 72% to 21% after the introduction of clinical practice guideline in a tertiary care pediatric hospital in Kansas city, Missouri, USA[36].

In the present study we further investigated factors that may be associated with the health facility administering ceftriaxone. Factors that were significant predictors of ceftriaxone administration included history of prior medication before admission to this hospital, patient having bacterial infection, being in surgical and medical wards. Patients with previous antibiotic use from another hospital were more likely to be provided with ceftriaxone by the health facility[37] possibly due to the fact that clinicians might have thought that the referred patients had to be put on a broad-spectrum regimen like ceftriaxone to cover for bacteria that might have resisted in the previous antibiotic exposure [38]. Patients having a presumptive diagnosis of bacterial infection significantly predicted ceftriaxone use. The reason could be that unavailability and inaccessibility to microbiological tests influence clinicians to use broad spectrum antibiotics to suppress infection and hence avoid prolonged illness[39].

Type of wards the patient was admitted in appeared to be associated with ceftriaxone use. Surgical 2 and Medical 1 wards are used for admissions for referral and new cases respectively. Surgical 2 ward usually admits postoperative patients; the fact that may explain the observed significant ceftriaxone administration.

A strength in the present study is that data were collected from a consultant and a tertiary care facility. This could imply and depict the real practice in many tertiary hospitals in Tanzania. However, we acknowledge inherent weaknesses of this study in that first, we could not ascertain treatment outcomes. Second, the data were strictly on the clinical acumen of the clinicians in deciding to prescribe ceftriaxone. Third, a poor documentation of the dosages and duration of ceftriaxone use in the patients’ files rendered it cumbersome for us to calculate the (Prescribed Daily Doses (PDDs) or (Defined Daily Doses (DDDs) which would have showed the volumes of ceftriaxone consumed. Third, there are other factors that come into play that this study could not investigate. These include a clinician prescription, pharmaceutical companies and health insurance companies’ influence. This could be an avenue for further study to explore the contribution of these factors and others toward excessive use of antibiotics in Tanzania.

## Conclusions

Ceftriaxone is extensively used in this hospital. We have observed inappropriately prolonged ceftriaxone surgical prophylaxis practice, empiric treatment with ceftriaxone particularly wound treatment with ceftriaxone without culture and sensitivity results. We recommend an antibiotic stewardship team to be instituted to constantly identify areas of improvement for optimal antibiotic use in general. The antibiotic stewardship team will also be instrumental to strike a balance between restricting antibiotic use to minimize antibiotic resistance, whilst allowing antimicrobial use where appropriate.
